# Cardiovascular magnetic resonance imaging of myocardial oedema following acute myocardial infarction: Is whole heart coverage necessary?

**DOI:** 10.1186/s12968-016-0226-5

**Published:** 2016-01-23

**Authors:** Stephen Hamshere, Daniel A. Jones, Cyril Pellaton, Danielle Longchamp, Tom Burchell, Saidi Mohiddin, James C. Moon, Jens Kastrup, Didier Locca, Steffen E. Petersen, Mark Westwood, Anthony Mathur

**Affiliations:** 1 Department of Cardiology, Barts Heart Centre, St Bartholomews Hospital, Barts Health NHS Trust, London, EC1A 7BE UK; 2 William Harvey Research Institute, NIHR Cardiovascular Biomedical Research Unit at Barts, Queen Mary University of London, Charterhouse Square, London, EC1M 6BQ UK; 3 Service de Cardiologie et Département de Médecine Interne, Centre Hospitalier Universitaire, Vaudois, Lausanne Switzerland; 4 Department of Cardiology, Rigshopitale, University of Copenhagen, Copenhagen, Denmark

## Abstract

**Background:**

AAR measurement is useful when assessing the efficacy of reperfusion therapy and novel cardioprotective agents after myocardial infarction. Multi-slice (Typically 10-12) T2-STIR has been used widely for its measurement, typically with a short axis stack (SAX) covering the entire left ventricle, which can result in long acquisition times and multiple breath holds. This study sought to compare 3-slice T2-short-tau inversion recovery (T2- STIR) technique against conventional multi-slice T2-STIR technique for the assessment of area at risk (AAR).

**Methods:**

CMR imaging was performed on 167 patients after successful primary percutaneous coronary intervention. 82 patients underwent a novel 3-slice SAX protocol and 85 patients underwent standard 10-slice SAX protocol. AAR was obtained by manual endocardial and epicardial contour mapping followed by a semi- automated selection of normal myocardium; the volume was expressed as mass (%) by two independent observers.

**Results:**

85 patients underwent both 10-slice and 3-slice imaging assessment showing a significant and strong correlation (intraclass correlation coefficient = 0.92;*p* < 0.0001) and a low Bland-Altman limit (mean difference −0.03 ± 3.21 %, 95 % limit of agreement,- 6.3 to 6.3) between the 2 analysis techniques. A further 82 patients underwent 3-slice imaging alone, both the 3-slice and the 10-slice techniques showed statistically significant correlations with angiographic risk scores (3-slice to BARI *r* = 0.36, 3-slice to APPROACH *r* = 0.42, 10-slice to BARI *r* = 0.27, 10-slice to APPROACH *r* = 0.46). There was low inter-observer variability demonstrated in the 3-slice technique, which was comparable to the 10-slice method (z = 1.035, *p* = 0.15). Acquisition and analysis times were quicker in the 3-slice compared to the 10-slice method (3-slice median time: 100 seconds (IQR: 65-171 s) vs (10-slice time: 355 seconds (IQR: 275-603 s); *p* < 0.0001.

**Conclusions:**

AAR measured using 3-slice T2-STIR technique correlates well with standard 10-slice techniques, with no significant bias demonstrated in assessing the AAR. The 3-slice technique requires less time to perform and analyse and is therefore advantageous for both patients and clinicians.

**Electronic supplementary material:**

The online version of this article (doi:10.1186/s12968-016-0226-5) contains supplementary material, which is available to authorized users.

## Background

Cardiac magnetic resonance (CMR) imaging has become the reference standard in the quantification of ventricular volumes, function and tissue characterisation [1]. T2 weighted imaging has been widely used in the assessment of myocardial oedema and area at risk (AAR) following an acute myocardial infarction (AMI) and has been hailed as a potential ‘gold standard’ [2]. The AAR is defined as the area of ischaemic myocardium that occurs distally to a coronary artery occlusion and its quantification has become crucial in assessing the efficacy of reperfusion therapy and novel cardioprotective agents. Additionally the AAR acts as a prognostic factor in patients following AMI and can play a role in decision-making regarding myocardial revascularization helping distinguish between necrosed and viable myocardium [3, 4].

Currently methods for assessing AAR require coverage of the whole left ventricle with the acquisition of 10–12 continuous myocardial short axis slices, with each slice acquired with a single breath hold of 10–15 seconds. This lengthens the overall duration of a CMR scan in patients early after a myocardial infarction and therefore techniques that shorten examination times may be advantageous to improve patient compliance. Furthermore, despite advances in semi-automated software, post processing and analysis of these images requires time-consuming manual analysis.

Our goal was to assess the AAR using 3 non-contiguous slices in comparison to conventional multi-slice contiguous slices in patients following successful primary percutaneous coronary intervention (PPCI) for acute myocardial infarction.

## Methods

Between April 2008 and November 2012, 167 patients with ST-segment elevation MI successfully reperfused through primary percutaneous coronary intervention (PPCI) and undergoing CMR within the first week after reperfusion were studied. All of these patients had been consented into interventional clinical trials including stem cell trial and pharmacological intervention trials (REGENERATE-AMI (NCT00765453), NITRITE-AMI (NCT01584453) and myocardial oedema in acute myocardial infarction (NCT00987259)) [5, 6]. These studies were approved by local ethics committee. Patients underwent either 3 or 10-slice T2 weighted imaging for the assessment of the AAR.

### CMR Protocol

Cardiac magnetic resonance (CMR) imaging was performed on a 1 · 5 T Philips Achieva scanner with a cardiac 32-channel phased array coil. Balanced steady-state free precession cine imaging was used to acquire 10–12 short axis slices (8 mm slice thickness, 2 mm gap) with one slice per breath-hold. Sequence parameters were 1.5 ms echo time (TE), 3.1 ms repetition time (TR), and acquired voxel size was 1.8 × 1.86 mm with a typical FOV of 360 mm in the phase encoding direction. We acquired 45 phases with 25 % phase sharing. Parallel imaging (SENSE) was used with an acceleration factor of 2.0.

**Fig. 1:**
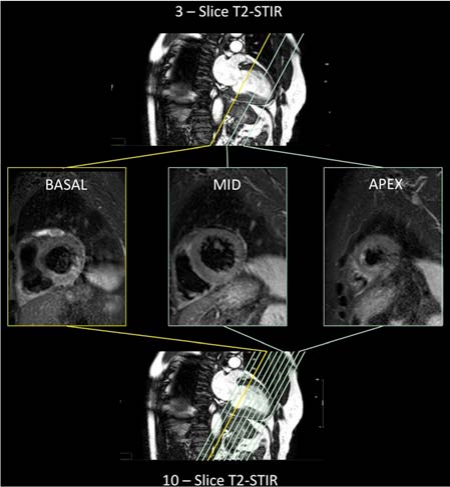
Acquisition protocol of the 3-slice and 10-slice T2-STIR techniques. Screenshot demonstrating the different acquisition protocol levels for basal, mid and apical slices in the 3-slice and 10-slice techniques

Myocardial oedema was assessed using fat suppressed T2-weighted triple inversion turbo spin echo STIR (Short tau inversion recovery) imaging (TE 80 ms, TR 2 heart beats, TSE factor 31, voxel size 1.8 ×1.8 mm). Either 10-slices (8 mm per slice, 2 mm gap matched to LGE/cine slices) or 3-slices (8 mm per slice, 19 mm gap with basal, midventricular and apical slices) with one slice per breath-hold (Fig. 1). This sequence has previously been used and validated for assessment of myocardial oedema and myocardial salvage index (MSI) [7–10].

Late gadolinium enhancement (LGE) images were acquired ten minutes after injection of a dose of 0 · 2 mmol/kg of gadoterate meglumine (Dotarem). A T1-weighted segmented inversion-recovery gradient echo pulse sequence (TR 3.9 ms TE 1.9 ms, flip angle 15°, voxel size of 2 × 2 mm, typical FOV 360 mm) was used to obtain 10–12 short axis slices (matched with the short-axis cine images) with one slice per breath-hold. The inversion time was adjusted individually according to a T1 scout sequence (Look-Locker). Images were acquired every other heart beat with 2 signal averages.

### Coronary angiography

All patients underwent coronary angiography according to local cardiac catheterisation laboratory protocols and standard primary percutaneous coronary intervention (PCI) with insertion of at least one stent after admission with a an acute STEMI with an ECG showing ST-segment elevation of 0.1 mV in two or more limb leads or 0.2 mV in two or more contiguous precordial leads, or presumed new left bundle branch block. Flow of the infarct related artery prior to PCI was characterized using the TIMI system (Thrombolysis in Myocardial Infarction) [11]. The AAR was established using angiographic risk scores (BARI and modified APPROACH scores [12, 13].

### Image analysis

All analysis was performed using a dedicated software reporting system (CVI^42^, Circle Cardiovascular Imaging Inc, Calgary, Alberta, Canada). All images were anonymised, batched and analysed in a blinded fashion by two experienced CMR operators. Manual endocardial and epicardial contours were drawn to calculate myocardial mass (MM), followed by semi-automated selection of normal myocardium with the oedema volumes being expressed as a mass (%). Area at risk (myocardial oedema) was described as >2SD in signal intensity from remote normal myocardium [14]. If present, a central core of hypointense signal within the area of increased signal intensity (hemorrhagic infarction) was included in the AAR [15]. Increased signal intensity from the blood pool adjacent to the endocardium due to slow flow was excluded. Infarct size was calculated using the full-width half maximum method as previously described [16–18]. Infarct and oedema endocardial surface area (ESA) was the percentage of the endocardial enhancement against the total endocardial area. In case of discordance between operators, blinded review by a level III certified CMR reader was performed. If myocardial haemorrhage was present within the area of increased signal intensity it was included in assessment area [15].

### Angiographic risk scores

The angiographic area at risk was assessed by the Modified Bypass Angioplasty Revascularisation Investigation [12] and the modified Alberta Provincial Project for Outcome Assessment in Coronary Heart Disease [APPROACH] [13] jeopardy scores). Coronary angiograms were reviewed by two experienced observers blinded to CMR scan and clinical data.

### Interobserver variability

Each AAR calculation method was assessed with the evaluator blinded to the results of other techniques. All studies, both angiographic and CMR, were evaluated separately by 2 cardiologists specialized in cardiac imaging and 2 interventional cardiologists to obtain the interobserver variability of each AAR estimation method.

### Statistical analysis

Baseline demographics and continuous variables are summarized for 3-slice and 10-slice groups. Continuous variables are presented as a mean ± SD and categorical variables are presented as a percentage. 95 % confidence intervals (CI) are given. Intra-observer variability and correlation between methods was calculated using the coefficient of intraclass correlation coefficient (ICC). All p-values are 2-sided and a value of <0.05 was considered to indicate statistical significance. The comparison of the different correlation coefficients was performed using a 2-tailed Fisher’s z-transformation statistical analysis. Data plotting used in analyzing the agreement between the different methods was made with Bland-Altman analysis [19]. All statistical analyses were performed using SPSS version 19 (IBM Corp. Armonk, NY, USA) and graphs produced using Graphpad Prism version 5.0 (GraphPad Software, San Diego, CA).

## Results

167 patients presenting with AMI undergoing PPCI at 3 European cardiac intervention centre {Barts Heart Centre, UK; Centre Hospitalier Universitaire, Switzerland; Rigshospitalet, Denmark), were included in the analysis. The mean ages of patient was 56.2 ± 10 · 2 years and 88 % were male. Clinical, CMR and angiographic characteristics of the population are shown in Table 1. Patients underwent either 10-slice (*n* = 85) or 3-slice (*n* = 82) T2-STIR imaging for AAR assessment as previously described (Additional file 1: Figure S1). The 2 groups were similar with regards to age, sex, LVEF and medical therapy, due to the inclusion criteria of the individual clinical studies there were a greater number of left anterior descending artery (LAD) occlusions in the 3 slice group. CMR was performed at a median of 3 days (range: 2–3 days) after PPCI. In all cases, increased signal intensity was detected in T2-STIR as well as in late gadolinium enhancement sequences.

**Table 1:** Baseline characteristics

	3-slice	10-slice	
	(*n* = 82)	(*n* = 85)	*P* value
Age (yr)	57.0 ± 10.5	55.9 ± 11.4	0.6202
Sex (M/F)	71/11	75/12	0.3037
BMI (kg/m^2^)	27.0 ± 3.8	27.4 ± 3.9	0.5277
Ethnicity (Caucasian) (No. (%))	66 (80 %)	71 (83.5 %)	0.6113
Medical History:			
Hypertension (No. (%))	30 (36.5 %)	28 (32.9 %)	0.6235
Hypercholesterolemia (No. (%))	24 (29.3 %)	31 (26.4 %)	0.3251
Diabetes mellitus (No. (%))	10 (12.2 %)	9 (11.6 %)	0.5766
Active smoker (No. (%))	42 (51.2 %)	47 (55.3 %)	0.6004
Previous MI (No. (%))	1 (1.2 %)	2 (2.4 %)	0.5841
Previous PCI (No. (%))	1 (1.2 %)	3 (3.5 %)	0.3320
Family history (No. (%))	25 (30.5 %)	19 (22.4 %)	0.2354
Culprit Vessel:			<0.0001
LAD (No. (%))	82 (100 %)	23 (27 %)	
LCx (No. (%))	0 (0 %)	11 (13 %)	
RCA (No. (%))	0 (0 %)	51 (60 %)	
Timings:			
Chest Pain to PCI (min)	194.5 ± 25.4	201.0 ± 32.2	0.8459
Infarct Size (%)	17.01 ± 8.88	18.02 ± 8.82	0.4543
AAR (%)	27.9 ± 8.3	27.27 ± 7.3	0.9800

Plus-minus values are mean ± SEM. No denotes number

*BMI* body mass index, *AAR* area at risk, *LAD* left anterior descending artery, *LCx* circumflex artery, *RCA* right coronary artery

### Comparison of 3-slice and 10-slice AAR in the 10-slice cohort

**Fig. 2:**
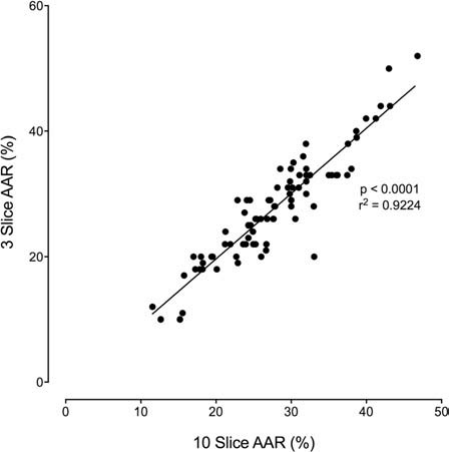
Correlation coefficient between 3-slice AAR and 10-slice AAR. Association between 3-slice STIR area at risk and 10-slice STIR area at risk assessed by CMR on sub group 10-slice STIR group

The 10-Slice AAR group underwent both 10-slice and 3-slice analysis to directly compare the 2 techniques. When assessing the 3-slice acquisition in the 10-slice cohort, all scans had the same level basal, mid and apical slice assessed (defined as 2nd basal slice after the aortic valve, 2nd slice with presence of papillary muscles defined as the mid ventricle and penultimate slice within the apex). Within the 10-slice AAR group the AAR ranged from 11.5 % of the myocardium to 46.8 % (mean 27.8 ± 7.3) and the 3-slice AAR assessment of the same patients showed similar values ranging from 10.0 to 52.0 % (mean 27.9 ± 8.3) (*p* = 0.9800). There were strong correlations between 3-slice AAR and 10-slice AAR in this patient group (*r* = 0.92, *p* < 0.0001) (Fig. 2). Bland-Atman assessment of the two assessment groups shows a low limit of agreement (mean difference −0.03 ± 3.21 %, 95 % limit of agreement, −6.3 to 6.3, containing 95.3 % (81/85) of the difference scores). In addition there was a strong correlation between 3-slice MM and 10-slice MM (*r* = 0.90, *p* < 0.0001) (Additional file 2: Figure S2).

### Comparison of CMR assessed AAR size and angiographic AAR

**Fig. 3:**
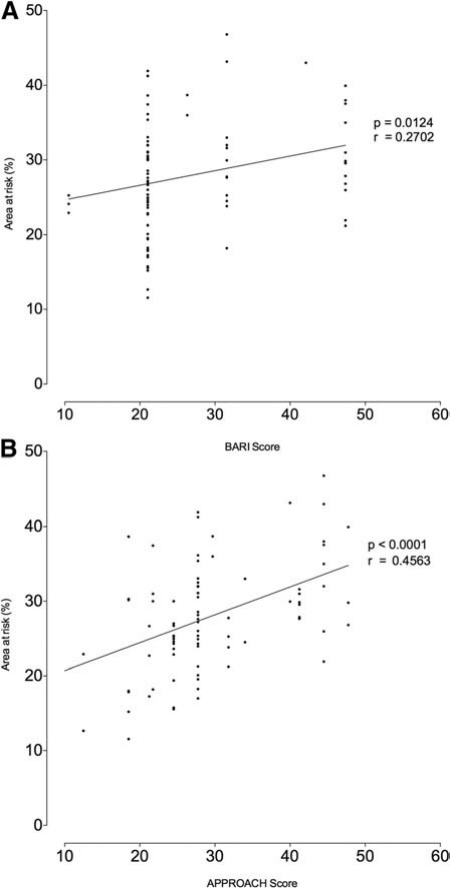
Scatter-plot of angiographic risk score versus 10-slice STIR AAR. Panel **a** shows the relationship between 10-slice STIR area at risk assessed by CMR and BARI angiographic risk. Panel **b** shows the relationship between 10-slice STIR area at risk assessed by CMR and APPROACH angiographic risk

Angiographic risk assessment was performed for all patients in this study. In the 10-slice AAR group the BARI angiographic risk score ranged from 10.5 % to 47.4 % (mean 26.6 ± 10.1), and the APPROACH angiographic risk score ranged from 6.5 % to 47.8 % (mean 29.2 ± 9.0 %) of the left ventricle (LV) myocardium. There was good correlation between the 2 angiographic risk scores (*r* = 0.85, *p* < 0.0001). There was a statistically significant correlation with discrepant strength between the 10-slice AAR and the APPROACH angiographic risk score (*r* = 0.46, *p* < 0.0001) with a weaker correlation seen between the 10-slice AAR and BARI angiographic risk score (*r* = 0.27, *p* = 0.0124) (Fig. 3). For the 10-slice AAR group infarct and oedema ESA was assessed for all patients, with Infarct ESA ranging from 0 to 45 % (mean 17.3 ± 9.8 %) and oedema ESA ranging from 10 to 44 % (mean 27.2 ± 7.8 %). There was a strong correlation between 10-slice AAR and oedema EAS (*r* = 0.79, *p* < 0.0001), there was a low correlation between 10-slice AAR and infarct EAS (*r* = 0.42, *p* < 0.0001).

**Fig. 4:**
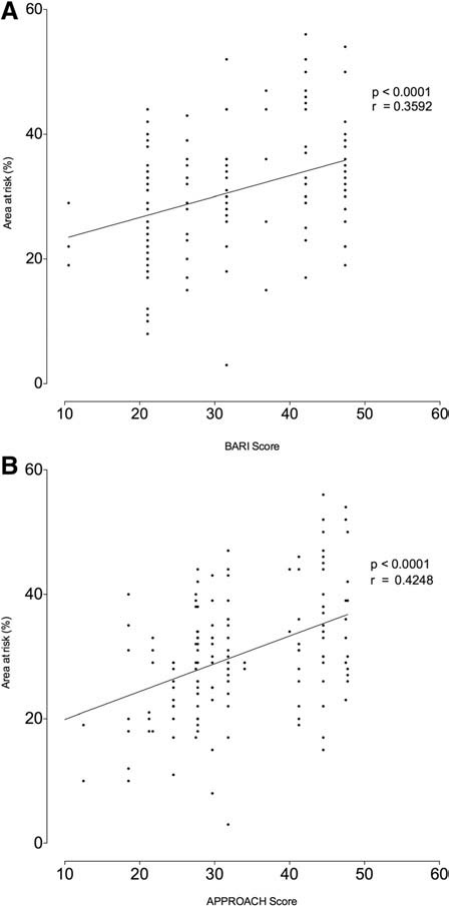
Scatter-plot of angiographic risk score versus 3-slice STIR AAR. Panel **a** shows the relationship between 3-slice STIR area at risk assessed by CMR and BARI angiographic risk. Panel **b** shows the relationship between 3-slice STIR area at risk assessed by CMR and APPROACH angiographic risk

In all 3-slice AAR studies the BARI angiographic risk score ranged from 10.5 to 47.4 % (mean 30.9 ± 10.5) and the APPROACH angiographic risk score ranging from 6.5 to 47.8 % (mean 33.4 ± 9.3 %) of the LV myocardium. As was seen within the 10-slice group there was a good correlation between the 2 angiographic risk scores (*r* = 0.88 *p* < 0.0001). Within the 3-slice AAR group the AAR ranged from 3 to 56 % (mean 30.1 ± 9.8). The 3-slice AAR assessment was correlated with both angiographic risk scores (BARI: *r* = 0.36, p <0.0001, APPROACH; *r* = 0.42, *p* < 0.0001) (Fig. 4) (Table 2). For the 3-slice AAR group infarct and oedema ESA was assessed, with infarct ESA ranging from 1 to 57 % (mean 17.3 ± 9.7 %) and oedema ESA ranging from 2 to 59 % (mean 29.1 ± 9.9 %). There was a strong correlation between 3-slice AAR and oedema EAS (*r* = 0.89, *p* < 0.0001), there was a low correlation between 3-slice AAR and infarct EAS (*r* = 0.45, *p* < 0.0001).

**Table 2:** Intraclass correlation coefficients between angiographic and cardiovascular magnetic resonance methods of 3-slice and 10-slice oedema CMR (T2-STIR) imaging techniques

3-slice Assessment	T2 STIR	APPROACH	BARI
Infarct ESA	0.50 (0.30–0.66)^***^	0.27 (0.12–0.41)^**^	0.19 (0.05–0.34)^**^
Oedema ESA	0.91 (0.86–0.95)^***^	0.36 (0.22–0.49)^**^	0.27 (0.12–0.41)^*^
Infarct %	0.47 (0.26–0.64)^***^	0.28 (0.13–0.41)^**^	0.20 (0.05–0.35)^*^
BARI	0.35 (0.21–0.49)^***^	0.88 (0.85–0.91)^***^	
APPROACH	0.42 (0.29–0.55)^***^		
10-slice Assessment	T2 STIR	APPROACH	BARI
Infarct ESA	0.64 (0.50–0.75)^***^	0.44 (0.25–0.60)^***^	0.28 (0.07–0.47)^**^
Oedema ESA	0.89 (0.84–0.93)^***^	0.40 (0.20–0.56)^***^	0.17 (–0.05–0.37)
Infarct %	0.73 (0.61–0.82)^***^	0.50 (0.32–0.64)^***^	0.33 (0.13–0.51)^**^
BARI	0.27 (0.06–0.46)^***^	0.85 (0.78–0.90)^***^	
APPROACH	0.46 (0.27–0.61)^***^		

Data are expressed intraclass correlation coefficients (confidence interval). *p* value <0.05 = *, <0.01 = **, <0.001 = ***. *APPROACH* Alberta provincial project for outcome assessment in coronary heart disease, *BARI* bypass angioplasty revascularization investigation myocardial jeopardy index, *ESA* endocardial surface area, *STIR T*2, short tau inversion recovery, *LAD* left anterior descending artery

### Comparison between infarct size and myocardial AAR Scores

**Fig. 5:**
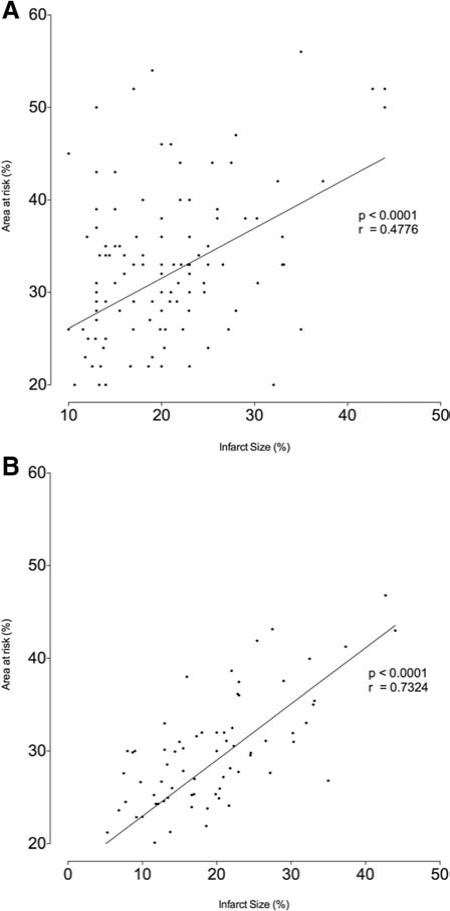
Correlation coefficient between infarct size and AAR. Panel **a** shows the association between 3-slice STIR area at risk assessed by CMR and infarct size. Panel **b** shows the association between 10-slice STIR area at risk assessed by CMR and infarct size

**Fig. 6:**
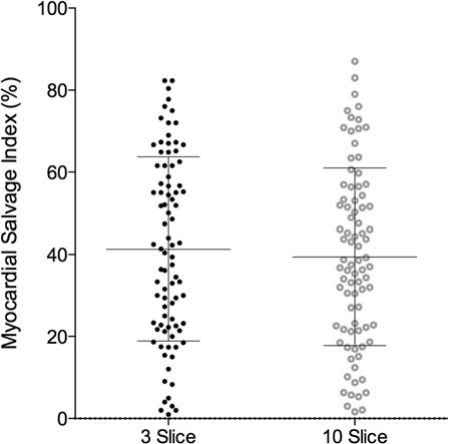
Myocardial salvage assessment between 3-Slice and 10-Slice technique. Panel **a** shows the relationship between 3-slice STIR area at risk assessed by CMR and infarct size. Panel **b** shows the relationship between 10-slice STIR area at risk assessed by CMR and infarct size

Infarct size as measured by CMR ranged from 1.0 % to 44.0 % of the LV myocardium (mean 18.0 ± 8.8 %). There was a significant correlation between infarct size and 10-slice AAR (*r* = 0.73, *p* < 0.0001), with a lower correlation seen between infarct size and 3-slice AAR studies (*r* = 0.48 *p* < 0.0001) (Fig. 5). Myocardial salvage was calculated for both 3-slice and 10-slice assessments with no significant difference between the two imaging modalities (41.30 % ± 22.41 % in the 3-slice group vs. 39.39 % ± 21.59 % in the 10-slice group; *p* = 0.5712) (Fig. 6).

### Timing for acquisition and analysis of 3-slice versus 10-slice AAR

**Fig. 7:**
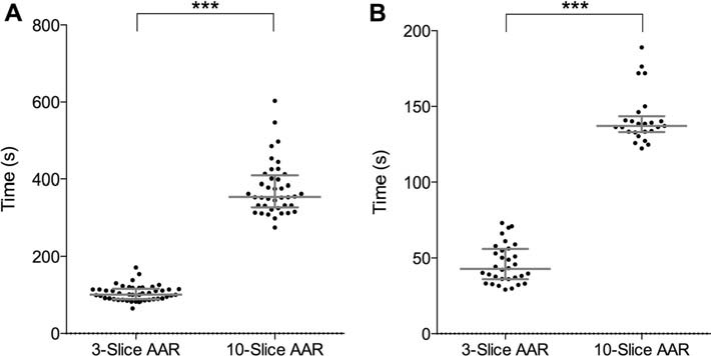
Comparison of acquisition **a** and analysis **b** time between 3-slice and 10-slice imaging technique. Panel **a** shows the relationship between 3-slice STIR area at risk assessed by CMR and infarct size. Panel **b** shows the relationship between 10-slice STIR area at risk assessed by CMR and infarct size

The time taken for the acquisition of the T2-STIR sequences was assessed from the initiation of the scan to the start of the next imaging sequence. The 3-slice group had a median acquisition time of 100 seconds (range: 65–171 seconds) and the 10-slice group had a median acquisition time of 355 seconds (range: 275–603 seconds), with a significant difference seen between the two groups (*p* < 0.0001) (Fig. 7a).

For analysis the 3-slice group had a median time of 43 seconds (IQR: 36–56 seconds) and 10-slice group had a median time of 137 seconds (IQR: 133–144 seconds), with a significant difference seen between the two groups (*p* < 0.0001). The inter-observer timing variability correlated well between analysers; 3-slice *r* = 0.96 *p* < 0.0001, 10-slice *r* = 0.93 *p* < 0.0001) (Fig. 7b).

Combined acquisition and analysis time showed a significant difference between the two groups (*p* < 0.0001). The median time of 143 seconds (IQR: 131–171 seconds) in the 3-slice group was on average 5 minutes quicker than the 10-slice group (median time of 492 seconds (IQR: 472–565 seconds).

Overall scan time was taken from initiation of first scouting image to the end of the final image. Within the 3-slice group there was a median time of 2163 seconds (IQR: 1740–2520 seconds) and 10-slice group had a median time of 2448 seconds (IQR: 1986–2835 seconds), with a significant difference seen between the two groups (*p* = 0.0057).

### Inter-observer variability

**Fig. 8:**
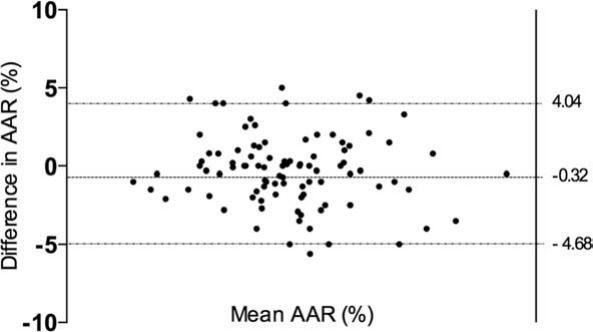
Bland-Altman plot for the inter-observer variability of the 3-slice STIR AAR assessment

3-slice STIR imaging showed low inter-observer variability. For the Bland-Altman plot, the 95 % limits of agreement (−5.3 %, 3.6 %) contained 95.1 % (78/82) of the difference scores. The mean bias of the measurements between observers was 0.08 %, and the maximum and minimum difference was 8.0 % and −7.0 % respectively (Fig. 8). The 10-slice STIR imaging showed similar inter-observer variability (z = 1.035, *p* = 0.15). For the Bland-Altman plot, the 95 % limits of agreement (−4.68 %, 4.04 %) contained 94.1 % (80/85) of the difference scores. The mean bias of the measurements between observers was −0.32 %, and the maximum and minimum difference was 5.0 % and −5.6 % respectively.

## Discussion

Oedema CMR imaging using T2-STIR remains an important technique to quantify the AAR. The aim of this study was to validate a 3-slice oedema CMR quantification of the myocardial AAR following PPCI for STEMI. We demonstrated that 3-slice quantification remains accurate for the detection of myocardial oedema after an AMI when compared to the conventional 10-slice technique with no significant bias demonstrated in assessing the AAR. The 3-slice technique also requires less time to acquire and analyse compared to the conventional 10-slice approach.

Oedema CMR using T2-STIR imaging remains popular for assessing myocardial oedema [20] and the AAR after AMI, with studies showing the superiority of the clinical use of oedema CMR imaging in both AMI and chronic heart disease [4, 21]. The benefit of oedema CMR assessment over previous AAR assessment such as single-photon emission computed tomography (SPECT) is the lack of ionising radiation or the need for tracer administration. Although the benefit of SPECT imaging processes removes the issues of claustrophobia that is found in CMR imaging. The majority of these studies have used 10–12 slice oedema CMR covering the whole left ventricle, however due to the need for adequate breath holding techniques and the possibility of artefact the overall image acquisition time is long for the 10-slice STIR imaging technique.

Previous studies have demonstrated the suitability of 3-slice imaging in the assessment of myocardial scar as well as LV volumes [22]. However this is the first study to assess whether or not the AAR can also be assessed using a 3-slice technique, demonstrating the technique is comparable to the conventional 10-slice approach. The main benefit of the 3-slice oedema CMR in comparison to 10-slice oedema CMR is a quicker acquisition and analysis time. The overall scan time was nearly 5 minutes shorter with the 3-slice oedema sequence compared to the longer 10-slice sequences. The difference of over 5 minutes due to a shorter acquisition time and analysis time would have clinical benefit. The use for only 3 breath holds in comparison to the 10 or 12 without loss of required clinical information is of benefit especially in patients who have issues including claustrophobia or who are unable to lie flat for extended periods. These are common factors in patients 3 days post primary PCI for AMI. The benefit that could be applied to patients could also be seen finically by performing 3-slice AAR in the clinical setting. In a busy CMR centre where multiple clinical scans are performed each day the reduction of acquisition time could result in additional scans being performed and improved financial incentive. In addition the timing for analysis was significantly shorter within the 3-slice approach without the loss of significant clinical information, which may be of benefit when multiple analysis are required thus reducing analysis times in large quantity analysis.

The assessment of AAR has become increasingly important when testing potentially cardioprotective therapies. Conventional imaging techniques in such trials tend to assess changes in left ventricular ejection fraction for their surrogate endpoint that require multiple visits. The use of a single AAR image acquisition at an early time point could possibly reduce the need for multiple scans and give early results without the need for protracted follow up. Although more information maybe required in the research setting the shorter image analysis times could have be an important factor when assessing multiple images within a study.

One particular issue with T2-STIR imaging is the false signal that occurs in area of blood pooling especially in patients with poor LV function [23]. In the apical segments of the LV there is increased blood pooling causing greater sub-endocardial bright artefact during myocardial quantification. The imaging technique for 10-slice acquisition takes single contiguous 1 cm slices from mid atria to true apex, whereas the 3-slice acquisition takes a slice in the basal, mid and apical segment of the LV therefore reducing true apical blood pooling (Additional file 3: Figure S3). The observer variability was similar in the 3 slice and 10-slice acquisition groups. This implies that the use of 3-slice acquisition imaging may not result in the loss of important clinical information and may reduce the issue of increased signal from pooled blood in comparison to 10-slice acquisition. Although T2-STIR imaging has been used widely other methods or programming techniques are available to reduce acquisition time such as navigator gated imaging technique and the use of early gadolinium enhancement that could be beneficial in the clinical setting.

In summary the advantages of 3-slice oedema CMR imaging over 10-slice imaging in assessing myocardial AAR are: 1) equal clinical information; 2) reduced imaging protocol time; 3) reduced breath holding time; and 4) reduced combined acquisition and analysis time.

### Study limitations

The present study was performed on a small limited patient population in patients who after suffered a STEMI and underwent successful PPCI with the majority of patients receiving a novel cardioprotective intervention at the time prior to undergoing CMR imaging within 3 days. One of the studies included in the analysis recruited only patients with anterior infarcts due to occlusions of the LAD which may have lead to bias in the results.

## Conclusions

This study was able to validate 3-slice oedema CMR quantification of AAR showing good correlations with current full coverage imaging techniques. The quicker and easier analysis method is advantageous for both patients and clinicians and could shorten acquisition time in patients who are either claustrophobic or have difficulty with breath holding technique.

## Additional files

Additional file 1: Figure S1. Consort diagram. Flow chart of study design summarizing flow of patients through study. (JPG 25 kb)

Additional file 2: Figure S2. Correlation coefficient between 3-slice MM and 10-slice MM. Association between 3-slice MM quantification and 10-slice MM quantification assessed by CMR. (TIFF 114 kb)

Additional file 3: Figure S3. Difference between T2-STIR distributions after myocardial infarction. Screenshot demonstrating the increased signal seen in the basal, mid and apical slices in a T2-STIR imaging technique after a ST elevation myocardial infarction involving each of the major epicardial coronary arteries. (JPG 88 kb)
