# Supplementary figures and images for: Cardiovascular magnetic resonance imaging of myocardial oedema following acute myocardial infarction: Is whole heart coverage necessary?
Source: J Cardiovasc Magn Reson. 2016 Jan 23;18:7. doi: 10.1186/s12968-016-0226-5 (PMC4724400; doi:10.1186/s12968-016-0226-5)

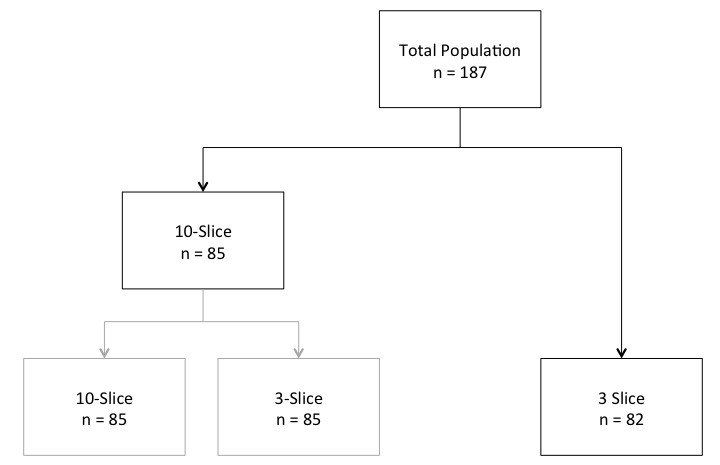

Supplement: Supplementary file 1 — Consort diagram. Flow chart of study design summarizing flow of patients through study. (JPG 25 kb) [file 12968_2016_226_MOESM1_ESM.jpg]

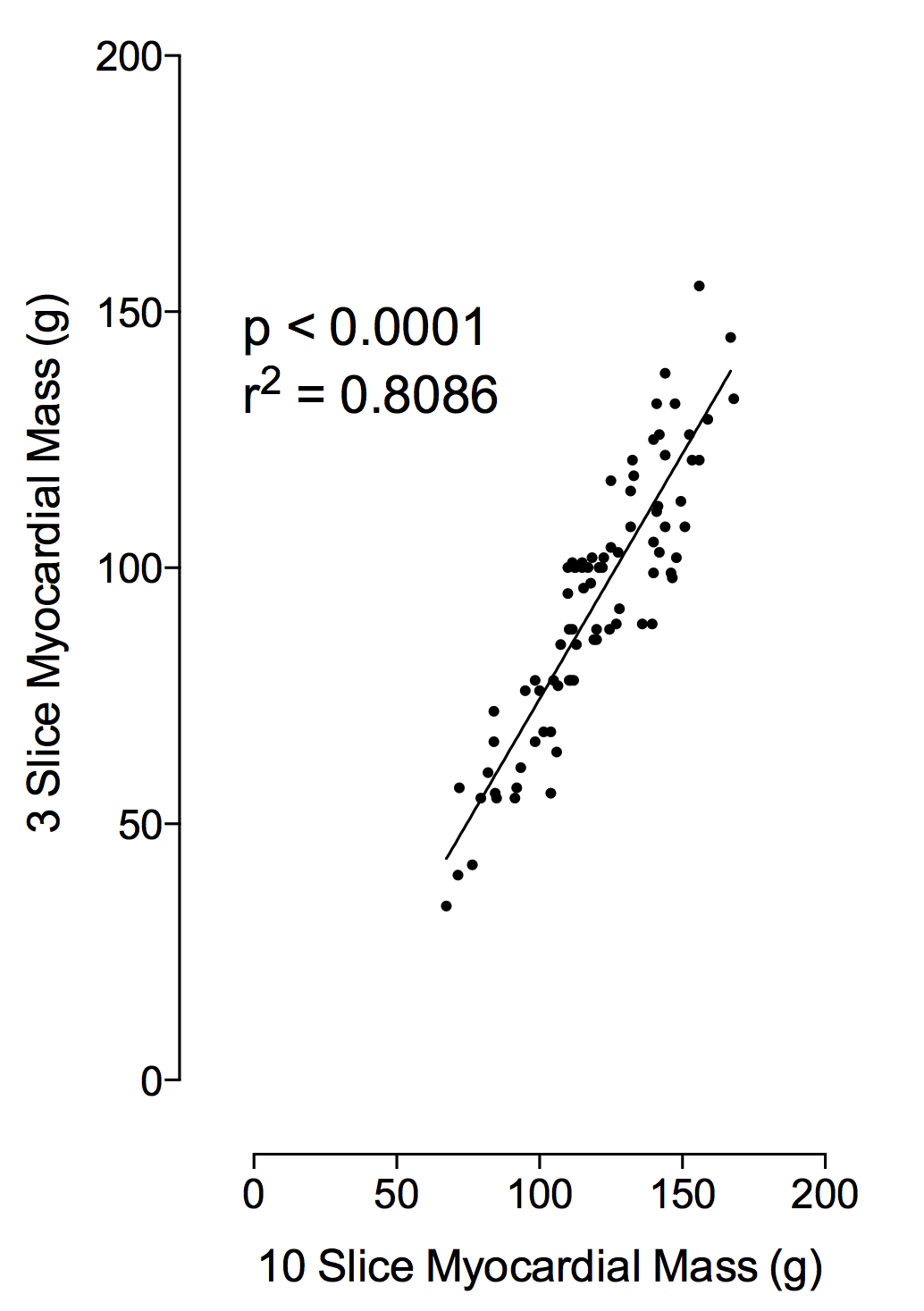

Supplement: Supplementary file 2 — Correlation coefficient between 3-slice MM and 10-slice MM. Association between 3-slice MM quantification and 10-slice MM quantification assessed by CMR. (TIFF 114 kb) [file 12968_2016_226_MOESM2_ESM.tiff]

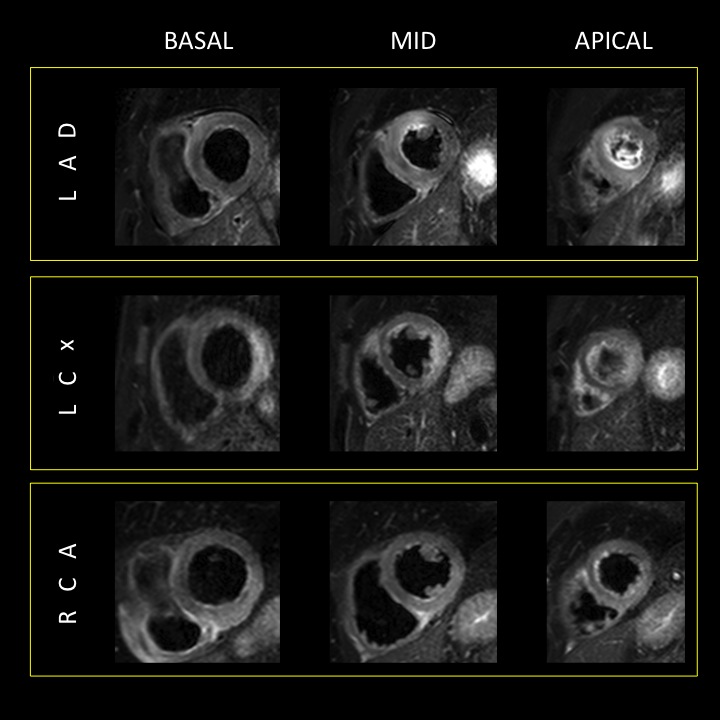

Supplement: Supplementary file 3 — Difference between T2-STIR distributions after myocardial infarction. Screenshot demonstrating the increased signal seen in the basal, mid and apical slices in a T2-STIR imaging technique after a ST elevation myocardial infarction involving each of the major epicardial coronary arteries. (JPG 88 kb) [file 12968_2016_226_MOESM3_ESM.jpg]
